# Quantitative Proteomics Reveals the Relationship between Protein Changes and Volatile Flavor Formation in Hunan Bacon during Low-Temperature Smoking

**DOI:** 10.3390/foods13091360

**Published:** 2024-04-28

**Authors:** Huiyu Zou, Chuangye Deng, Junnian Li, Aihua Lou, Yan Liu, Jie Luo, Qingwu Shen, Wei Quan

**Affiliations:** 1 College of Food Science and Technology, Hunan Agricultural University, Changsha 410128, China; 15088039856@163.com (H.Z.); d15700760840@163.com (C.D.); jieaccept@163.com (J.L.); louaihua916@163.com (A.L.); 18707491871@163.com (Y.L.); luojie@hunau.edu.cn (J.L.); 2 State Key Laboratory of Food Science and Resources, Jiangnan University, Wuxi 214122, China

**Keywords:** Chinese bacon, Hunan bacon, quantitative proteomics, volatile flavor compounds, protein degradation

## Abstract

This study aimed to investigate the changes in proteins and volatile flavor compounds that occur in bacon during low-temperature smoking (LTS) and identify potential correlations between these changes. To achieve this, a combination of gas chromatography-mass spectrometry and proteomics was employed. A total of 42 volatile flavor compounds were identified in the bacon samples, and, during LTS, 11 key volatile flavor compounds with variable importance were found at a projection value of >1, including 2′,4′-dihydroxyacetophenone, 4-methyl-2H-furan-5-one, Nonanal, etc. In total, 2017 proteins were quantified at different stages of LTS; correlation coefficients and KEGG analyses identified 27 down-regulated flavor-related proteins. Of these, seven were involved in the tricarboxylic acid (TCA) cycle, metabolic pathways, or amino acid metabolism, and they may be associated with the process of flavor formation. Furthermore, correlation coefficient analysis indicated that certain chemical parameters, such as the contents of free amino acids, carbonyl compounds, and TCA cycle components, were closely and positively correlated with the formation of key volatile flavor compounds. Combined with bioinformatic analysis, the results of this study provide insights into the proteins present in bacon at various stages of LTS. This study demonstrates the changes in proteins and the formation of volatile flavor compounds in bacon during LTS, along with their potential correlations, providing a theoretical basis for the development of green processing methods for Hunan bacon.

## 1. Introduction

Chinese pork bacon is one of the most representative traditional and popular meat products in China, known as “Larou” in Chinese [1]. Traditional Chinese pork bacon is now being introduced to the rest of the world. There are various types of traditional Chinese pork bacon in China, including Sichuan bacon, Yunnan bacon, Hunan bacon, and Jiangxi bacon [2]. Among these, Hunan bacon, produced in southern China, is preferred by local people for its unique and attractive smell, texture, and color. Although traditional Chinese bacon is mostly processed via prolonged marinating and smoking to allow the meat products to develop distinctive colors, flavors, and textures, there are significant differences in the smoking parameters used to prepare the different types of Chinese bacon; this is the main reason for the variation in flavors among different preserved meats [3]. As an example, bacon produced in other regions is smoked at 80 °C using smoking materials [4]. The traditional wood-burning smoking method of bacon production involves the use of smoke at temperatures of up to 500 °C [5]. In contrast, Hunan bacon in this study was subjected to intermittent low-temperature smoking at 5 °C and 55 °C for 4 h at each temperature in a continuous cycle for 10 days.

Many factors influence the overall acceptance of bacon, among which flavor is normally considered one of the most important qualities of bacon products [6]. In recent years, many quantitative studies have examined the volatile flavor substances in smoked bacon [7]. The degradation of lignin in the smoking liquid mainly produces aldehydes, and volatile flavor compounds dominated by aldehydes (10), phenols (10), ketones (13), and hydrocarbons (12) have been identified in smoked meat [5]. The flavor of bacon is closely related to the processing conditions, in addition to the action of the smoking liquid. The generation of volatile flavor compounds depends on various biochemical reactions in meat, such as lipid oxidation and degradation, protein degradation, and the Maillard reaction, among other pathways [8]. Li et al. reported that proteins in bacon undergo various dynamic changes during processing; they are first degraded into peptides and then further degraded into volatile flavor compounds, nonvolatile flavor substances, and free amino acids through the catalytic action of peptidase, which affects the quality of bacon and the formation of flavor [9]. During the processing of Xuan’en ham, myofibrillar and sarcoplasmic proteins are mainly degraded in the early stages of curing and fermentation; the products of protein degradation usually include branched-chain alcohols, aldehydes, ketones, and carboxylic acids in aromatic and ester substances [9]. During storage, the interactions between the free amino acids and volatile organic compounds in bacon play a key role in regulating the flavor of the final product, enhancing its organoleptic properties [10]. To date, limited research has been conducted on the relationship between volatile compounds and protein changes during the LTS process of Hunan bacon. Therefore, it is important to explore the formation of volatile flavor compounds in Hunan bacon during LTS.

Proteomic methods provide valuable information on the characteristics of proteins in meat products and the biological mechanisms occurring during processing. Many studies have applied proteomic technologies to the analysis of meat; the relationship between proteomics and flavor compounds has also been examined [11]. For example, proteomics was adopted to study the changes in the proteome of goat meat under different irradiation protocols, and 26 differentially expressed proteins related to odor were identified; these were mainly involved in the metabolism of cysteine and methionine and the oxidation of proteins [12], suggesting that proteomics can help discover biomarkers and predict the biochemical transformation of meat products [13].

Extensive research has been conducted on changes in flavor precursors and microbial growth, both of which affect flavor, in different types of bacon. However, there has been limited research on the changes in the proteome and volatile compounds, as well as their relationship with each other, during the LTS process of Hunan bacon. Gas chromatography-mass spectrometry (GC-MS) and multivariate analysis were employed to investigate the fluctuation pattern of volatile flavor compounds in bacon during LTS and to identify the most critical volatile flavor compounds in bacon that underwent LTS. Furthermore, label-free quantification (LFQ) was employed to investigate the alterations in the proteome of Xiangxi bacon throughout the curing process. The proteins associated with volatile flavor compounds were identified through correlation analysis, with the objective of elucidating the formation mechanism of volatile compounds in bacon during LTS. The results of this study are expected to provide a comprehensive explanation of how changes in proteins influence the formation of flavor substances during the LTS process of Hunan bacon, which may provide a theoretical basis for improving the quality of Hunan bacon.

## 2. Materials and Methods

### 2.1. Sample Preparation

Fresh pancetta was procured from six pigs carcass (Duroc pigs, weighing 100 ± 2 kg) at a local slaughterhouse in Xiangtan City (Hunan, China). The pork pate was divided into strips (length, 50–55 cm; width, 5–10 cm; thickness, 3–5 cm; weighing, 600 ± 50 g). These strips were then randomly divided into thirty-six equal groups for the production of Hunan bacon. The meat strips were cured with sodium chloride (3.5%, *w*/*w*), nitrite (0.01%, *w*/*w*), and D-isoascorbate sodium (0.15%, *w*/*w*) for 5 days at 4 °C. The baking temperatures were set at 55 °C and 5 °C, the humidity was set at 60%, and each temperature was maintained for 4 h, followed by the next temperature being maintained for 4 h. This round-trip cycle was repeated for 10 days. During this period, the fumigant was sprayed twice a day at regular intervals and in a quantitatively consistent manner. Bacon samples were collected at 0, 2, 4, 6, 8, and 10 days during the LTS process and stored at −80 °C for further analysis.

### 2.2. Measurement of Basic Index

The pH of the samples was measured using a digital pH meter equipped with a penetration probe (Testo 205, Titisee-Neustadt, Germany). The moisture content of the samples was measured according to the Chinese National Food Safety Standard [14]. The lightness (L*), redness (a*), and yellowness (b*) of the samples were measured on the surface using a CS-580A colorimeter (CHN Spec, Hangzhou, China). The texture profile of the samples was analyzed as described by Gao et al. [15]. Briefly, the samples were divided into sections of approximately 2 cm × 2 cm × 1 cm (length × width × height) and measured using a P/36R probe. The test parameters were as follows: pretest rate, 2.00 mm/s; test rate, 1.00 mm/s; return rate, 2.00 mm/s; compression ratio, 35%; and trigger point load, 5 g.

### 2.3. Electronic Nose (E-Nose) Analysis

A PEN3 E-nose system (AIRSENSE, Schwerin, Germany) was used to detect changes in the odors of bacon samples during LTS [16]. Before each measurement, the baseline was calibrated by running clean air from the instrument room until the relative conductivity of each probe was stabilized to approximately 1. Briefly, the minced bacon sample (4.0 g) was placed into a 15 mL headspace container and heated in a 60 °C water bath for 8 min. The internal flow rate was set to 400 mL/min, and the sample preparation, detection, auto-zero, and cleaning times were set to 5, 80, 5, and 80 s, respectively. The E-nose system contained 10 sensor probes: W1C (sensitive to aromatic compounds and benzene), W5S (highly sensitive to nitrogen oxides), W3C (sensitive to ammonia), W6S (sensitive to hydrides), W5C (sensitive to olefins and short-chain aromatic compounds), W1S (sensitive to methyl compounds), W1W (sensitive to sulfides and pyrazines), W2S (sensitive to sulfides and pyrazines), W2W (sensitive to organic sulfides), and W3S (sensitive to long-chain alkanes).

### 2.4. GC-MS Analysis of Volatile Compounds

All volatile compounds in the bacon sample were analyzed using an Agilent 7890B-5977 gas chromatograph equipped with headspace solid-phase microextraction (HS-SPME) technology and a quadrupole mass analyzer, based on a previously reported method with some modifications [17]. Each bacon sample (2.0 g) was minced and placed in a 15 mL headspace bottle to which *o*-dichlorobenzene (10 µL, 230 µg/mL) was added as an internal standard; then, the bottle was placed in a water bath at 60 °C for 30 min. The volatile compounds in the bacon samples were extracted for 30 min using an SPME fiber (50/30 µm, DVB/CAR/PDMS, Supelco, Bellefonte, PA, USA), after which the fiber was inserted into the GC-MS system. The volatile compounds were separated in a HP-5MS column (30 m × 0.25 mm, 0.25 µm, Agilent, Santa Clara, CA, USA) using helium as a carrier gas in splitless mode at a flow rate of 1.0 mL/min. The column temperature was initially maintained at 40 °C for 3 min, raised to 230 °C at a rate of 3 °C/min, and then maintained at 230 °C for 5 min. Electron impact ionization (70 eV) was used as the ion source at a temperature of 240 °C, detector voltage of 350 V, and scan range of 33–300 m/z in full-scan mode. The volatile compounds were identified through a comparison of the mass spectra with those in the NIST 17 Mass Spectral Library and confirmed via the retention index, which was calculated using a standard C6–C30 n-alkane series under the same chromatographic conditions. Semi-quantitative analysis was performed using the mass concentration of a standard internal substance (*o*-dichlorobenzene) and the peak areas of the internal standard and target substances.

### 2.5. Protein Degradation and Oxidation

To analyze protein degradation, the tricarboxylic acid (TCA)-soluble peptides of bacon samples were analyzed following a previously reported method with minor modifications [18]. The minced bacon sample (3.0 g) was homogenized with cold TCA solution (27 mL) at 10,000 rpm for 2 min, placed at 4 °C for 1 h, and then centrifuged at 12,000 rpm for 15 min. After centrifugation, the supernatant was collected for colorimetric analysis.

To measure protein oxidation, the total carbonyl and total sulfhydryl group contents of the bacon sample were measured as described in the literature, with appropriate modifications [19]. The contents of carbonyl and sulfhydryl groups were expressed as nanomoles of carbonyl per milligram of protein and nanomoles of sulfhydryl groups per milligram of protein, respectively.

### 2.6. Detection of Free Amino Acid

Amino acids were determined using high-performance liquid chromatography (HPLC) as described in our previous study [20]. First, minced bacon sample (100.0 mg) was ultrasonically extracted with 0.02 M HCl (10 mL) and then centrifuged at 14,000 rpm for 15 min. The supernatant was subjected to precolumn derivatization. Moreover, 10 µL of the supernatant was automatically derived with 5 mg/mL of *o*-phthalaldehyde and 10 mg/mL of 9-fluorenylmethyl chloroformate in an autosampler reaction vessel and mixed for 20 s before injection. The derived amino acids were separated and detected using an Agilent 1100 HPLC system (Santa Clara, CA, USA) equipped with a G1314A variable wavelength detector and an ODS HYPERSIL column (5 µm; 4.6 × 250 mm i.d.). The parameters used for HPLC analysis were the same as in our previous study. Agilent ChemStation software (10.0) was used for data acquisition and processing, and amino acid standards were used for quantification.

### 2.7. SDS-PAGE Analysis of Sarcoplasmic and Myofibrillar Proteins

Sarcoplasmic and myofibrillar proteins were extracted from the bacon sample using a previously reported method [21]. Changes in the molecular mass of sarcoplasmic and myofibrillar proteins in the samples were characterized using SDS-PAGE as described in the literature [22]. Briefly, 30.0 µg of protein sample was mixed with pH 6.8 loading buffer (0.13 M Tris, 2% SDS, 5% β-mercaptoethanol, 8% glycerol, 0.002% bromophenol blue) and desaturated by heating at 100 °C for 5 min prior to electrophoresis. Then, the sample was denatured and subjected to reducing SDS-PAGE (4% stacking gel and 12% separating gel) at a constant current of 30 mA using a BioRad Mini-Protean III dual slab cell (Hercules, CA, USA). After electrophoresis, the gels were stained with 0.2% Coomassie Brilliant Blue (G 250) for 1 h and destained using a decolorizing solution (methanol/acetic acid/water, 20:30:50, *v*:*v*:*v*).

### 2.8. LFQ

#### 2.8.1. Protein Extraction and Digestion

The sample was thoroughly mixed in 400 µL of RIPA working liquid, and a steel ball was added to perform the low-temperature grinding process (70 Hz, 4 min). The homogenate was centrifuged at 4 °C and 12,000 rpm for 10 min and transferred to a 1.5 mL Eppendorf tube. Ultrasound was performed in an ice water bath using an ultrasonic cell disruption system (JY96-IIN, Changzhou, China) for 20 min. After the sample was sufficiently ground and centrifuged (12,000 rpm, 4 °C, 10 min), the supernatant was collected and stored at −80 °C. The protein concentration was determined using the bicinchoninic acid protein quantitation method.

Protein samples (100.0 μg) were taken from the supernatant mixture, diluted with RIPA lysate to ~1 mg/mL, mixed with five volumes of precooled acetone, and precipitated overnight at −20 °C. The precipitate was centrifuged (12,000 rpm, 4 °C, 10 min), rinsed twice with 80% acetone, and centrifuged again. Then, 100 µL of protein solution was remixed, dissolved, and precipitated in a water bath for 3 min. Dithiothreitol (5 mM) was added and the solution was incubated at 55 °C for 20 min to reduce the disulfide bonds. After the sample was cooled to room temperature, iodoacetamide was added to a final concentration of 15 mM, and the reduced disulfide bonds were alkylated for 30 min while being protected by light. The protein was digested with trypsin by thorough mixing at a trypsin/protein ratio of 1:50 and incubated at 37 °C and 1000 rpm overnight. Trifluoroacetic acid was added to the mixture (to a final concentration 2%), and the sodium deoxycholate was thoroughly mixed and precipitated. After high-speed centrifugation for 10 min, the supernatant was collected. Then, 300 mL of 2% trifluoroacetic acid was added, mixed thoroughly, and centrifuged at 12,000 rpm for 10 min, and the coprecipitated polypeptide was extracted; the extraction was repeated twice. The polypeptide sample was obtained by combining the supernatant several times and performing high-speed centrifugation for 10 min. The peptide was desalted using a C18 column (PePSep C18, 1.9 µm × 75 µm × 15 cm, Bruker, Germany), and the digested sample was stored at −80 °C.

#### 2.8.2. Nano LC-MS/MS Analysis

Total peptides (200.0 mg) were separated from each sample using nanoElute2, a nano-UPLC liquid phase system, and data were collected using tims-TOF-Pro2, a mass spectrometer equipped with a nanoliter ion source. Separation was performed on a reverse-phase column (PePSep C18, 1.9 µm, 75 µm × 15 cm, Bruker, Germany). Mobile phase A was 0.1% formic acid aqueous solution, and mobile phase B was 0.1% formic acid acetonitrile solution. After the column was equilibrated to 100% phase A, the samples were directly loaded into the column by the automatic injector and then separated by the column gradient with a flow rate of 300 nL/min and gradient duration of 60 min. The mobile phase B ratio was as follows: 2% for 0 min, 2–22% for 45 min, 22–37% for 5 min, 37–80% for 5 min, and 80% for 5 min. The mass spectrometer adopted the DDAPaSEF mode for data-dependent acquisition, and the scanning range was 100–1700 m/z. During PASEFMS/MS scanning, the impact energy increased linearly with ionic mobility, from 20 eV (1/K0 = 0.6 Vs/cm^2^) to 59 eV (1/K0 = 1.6 Vs/cm^2^).

### 2.9. Statistical Analysis

All analyses were performed in triplicate, and the data were expressed as mean and standard deviation. SPSS ANOVA was used for statistical and significance analyses of the obtained data (*p* < 0.05); Origin 12.0 and GraphPad Prism 8 were used for data mapping. Differences were considered statistically significant at *p*-values of <0.05 or <0.01. SIMCA 14.1 was used for principal component analysis (PCA) and partial least squares discriminant analysis (PLS-DA). MetaboAnalyst 5.0 online software was used for hierarchical cluster analysis. Proteins with a selection multiple change (fold change [FC]) of >1.2 or <0.83 were considered statistically significant.

## 3. Result and Discussion

### 3.1. Analysis of Physicochemical Properties

As shown in Table 1, L* and b* values increased significantly from 48.2 to 57.0 and from 5.28 to 10.25 (*p* < 0.05), respectively. Although the a* value decreased in the early stage of LTS, it was not significantly changed by the end of LTS. Additionally, the moisture content of bacon decreased significantly from 69.9% to 39.2% (*p* < 0.05) during LTS, which was similar to the results reported by Du et al. [23]. Textural parameters such as hardness, adhesion, chewiness, and elasticity of bacon samples also increased significantly during LTS compared to 0 d (*p* < 0.05).

The color and texture of bacon depends on many factors. Wu et al. found that the degree of oxidation of myoglobin in meat significantly affected meat color, with meat color changing from bright red to brown as the degree of oxidation increased. [24]. The considerable enhancement in L* and b* during LST (*p* < 0.05) in this study indicates that myoglobin in bacon is exposed to oxygen in the air, resulting in a color change. Furthermore, studies have indicated that the texture of meat is influenced by changes in water holding capacity as well as the strength of intermolecular binding of side chains between proteins and the degree of protein hydrolysis [25]. The results of the present study are comparable to those of previous studies, which have demonstrated that a reduction in the moisture content of bacon is accompanied by a significant increase in texture parameters (*p* < 0.05).

### 3.2. Changes in the Composition of Volatile Flavor Compounds in Bacon

**Figure 1:**
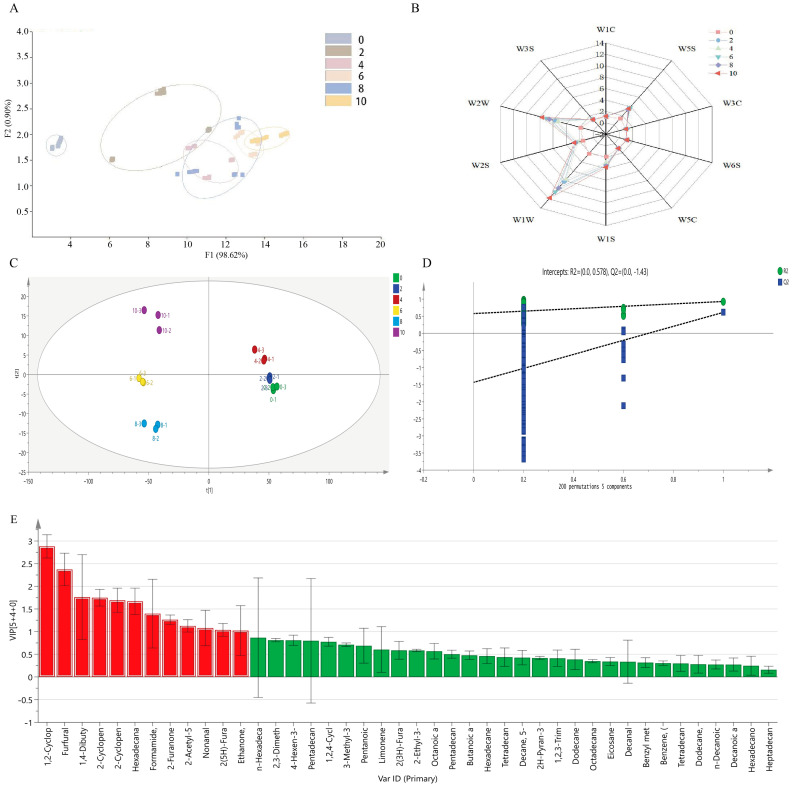
Changes in the composition of volatile flavor in bacon during LTS. (**A**) PCA of E-nose. (**B**) Radar map of E-nose. (**C**) OPLS-DA of GC-MS. (**D**) Cross-validations of the OPLS-DA model. (**E**) VIP plot of OPLS-DA.

As shown in Figure 1A, the reproducibility of parallel samples was analyzed via PCA, yielding a total score of 99.52%, which indicated that the model had a good ability to predict volatile compounds in bacon samples during different stages of LTS. The E-nose radar map (Figure 1B) illustrates the changes in the volatile flavor characteristics of Hunan bacon based on the signal values of 10 probes. Among them, the W5S, W1S, W2W, and W1W sensors were mainly sensitive to bacon samples and corresponded to nitrogen oxides; alkanes and aromatic compounds; organic sulfides; and sulfides, respectively, suggesting that bacon samples may contain high levels of compounds such as aldehydes, ketones, alkanes, and sulfides. E-noses have been widely used for the evaluation of food quality because they are rapid, sensitive, and nondestructive. This is analogous to previous findings that different types of wood-smoked bacon are equally susceptible to W2W, W5S, W2S, W1W, and W1S sensors and can be effectively distinguished from them [16].

E-nose analysis revealed significant changes in flavor during the LTS of bacon and that the type, content, and balance of volatile flavor compounds played a crucial role in impacting the flavor. A total of 42 volatile flavor compounds were identified in the bacon sample via SPME-GC-MS: 13 ketones, 11 hydrocarbons, 7 aldehydes, 5 acids, 4 esters, 1 furan, and 1 nitride (Table S1). Ketones were the most abundant (67.9%), followed by aldehydes (14.4%). Previous studies have shown that ketones and aldehydes are produced during amino acid breakdown and fat oxidation [26]. The content of the majority of volatile compounds underwent a significant change during LTS, particularly 3-methyl-1,2-cyclopentanedione, 3-methylcyclopent-2-en-1-one, and 2,3-dimethyl-2-cyclopentene-1-one, with the ketones exhibiting flavors that are commonly perceived as burnt and sweet. This indicates that low-temperature liquid smoking may enhance the flavor of bacon.

### 3.3. Screening of Key Volatile Flavor Compounds in Bacon during LTS

Multivariate statistical analysis of volatile flavor compounds in bacon samples was performed using a standardized operation method for automatic calculations. As shown in Figure 1C, the bacon samples from different stages of LTS were well separated in different regions of the OPLS-DA scatter plots, suggesting there were significant differences between the volatile flavor compounds of bacon samples. The parameters for the orthogonal PLS-DA (OPLS-DA) model were R2X = 0.998 and R2Y = 0.963, with a model prediction index (Q2) of 0.714. After 200 permutation tests (Figure 1D), the intersection of the Q2 regression line and the vertical axis was <0.0, indicating that the model did not exhibit overfitting and that its validation was effective.

To clarify the differences in bacon samples obtained from different stages of LTS, the volatile flavor compounds with variable importance in projection (VIP) values of >1.0 and statistically significant differences (*p* < 0.05) were defined as potential key volatile marker compounds that were considered to play a crucial role in the OPLS-DA discrimination process. A higher VIP value indicated a more significant contribution to the underlying variation. In Figure 1E, the red part of the VIP image shows the arrangement of differential metabolites with VIP > 1. In total, 11 volatile compounds (6 ketones, 3 aldehydes, 1 furan, and 1 nitride) were selected as differential volatile flavor compounds. Table 2 lists the volatile flavor compounds in bacon samples, which contribute significantly to the flavor of liquid smoked bacon.

Ketones and aldehydes are the main volatile flavor compounds in meat products and are present at lower thresholds, and they produce rich flavors during meat processing [27]. As shown in Table 2, the six ketones subjected to analysis in this study accounted for a significant proportion of the characteristic volatile compounds in bacon (76.4%). This included 2′,4′-dihydroxyacetophenone, 3-methylcyclopent-2-en-1-one, 4-methyl-2H-furan-5-one, 2-furanone, 2,5-dihydro-3,5-dimethyl, 3-methyl-1,2-cyclopentanedione, and 2,3-dimethyl-2-cyclopentene-1-one. Furthermore, three aldehydes were identified as volatile flavor compounds that could differentiate between bacon samples: furfural, nonanal, and hexadecanal. Other volatile compounds, including furans and nitrides, such as 2-acetyl-5-methylfuran and *N*,*N*-dibutylformamide, were also identified. The content of these differential volatile flavor compounds increased significantly during LTS (*p* < 0.05). It can be reasonably assumed that furfural and 3-methyl-1,2-cyclopentanedione have the greatest impact on flavor, given their relatively high VIP values. In previous studies, these volatile compounds were shown to be produced mainly during the oxidative denaturation of proteins and lipids. For example, ketones can be produced by lipid oxidation and amino acid metabolism [28], and aldehydes are produced in association with protein hydrolysis or amino acid degradation [29]. Moreover, it has been shown that furans are produced via the Maillard reaction [30]. It is therefore postulated that the enhancement of flavor during the LTS process may be influenced by the oxidative degradation of proteins.

### 3.4. Degradation and Oxidation of Bacon Proteins during LTS

The flavors of meat products are produced mainly through lipid and protein degradation, the Maillard reaction, and Strecker degradation. To understand the effect of LTS on the degradation of meat protein, the composition of myofibrillar and sarcoplasmic proteins and the contents of TCA-soluble peptides, free amino acids, total sulfhydryl, and carbonyl were analyzed.

#### 3.4.1. Protein Degradation

**Figure 2:**
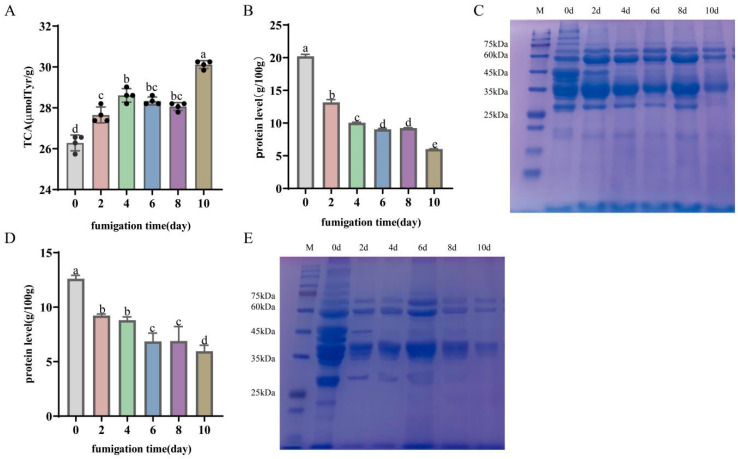
Protein degradation of Hunan bacon during LTS. (**A**) TCA of bacon during LTS. (**B**) Changes in myofibrillar protein during LTS. (**C**) SDS-PAGE of myofibrillar protein during LTS. (**D**) Change in myogen protein during LTS. (**E**) SDS-PAGE of myogen protein during LTS. ^a–e^ Means within rows and same breed with different superscripts differ significantly (*p* < 0.05).

As shown in Figure 2A, the content of TCA-soluble peptides significantly increased from 26.28 μmol tyr/g to 30.12 µmol tyr/g (*p* < 0.05), reflecting the greater extent of protein degradation at the end of LTS. A previous study showed similar results, with protein breakdown increasing with smoking duration [31]. Myofibrillar protein (MP) and sarcoplasmic protein (SP) are important components of pork proteins, accounting for 60–70% and 20–30%, respectively [32]. Therefore, to investigate protein degradation during LTS, this study further analyzed the changes in MP and SP during LTS (Figure 2B,D), revealing decreases from 20.21 g/100 g to 6.03 g/100 g and from 12.61 g/100 g to 5.96 g/100 g (*p* < 0.05), respectively. This indicated that MP and SP were degraded during LTS.

Similarly, SDS-DAGE results confirmed the degradation of MP and SP during LTS of bacon (Figure 2C,E). The electrophoresis bands in the 100, 75, 60, 45, 35, and 25 kDa regions show the degradation of MP (Figure 2C), potentially α-actin (96 kDa), actin (43.2 kDa), β-tropomyosin (35 kDa), TnT (32 kDa), MLC1 (27.1 kDa), and troponin C (20 kDa) [33]. As shown in Figure 2E, at the end of LTS, bands between 25 and 75 kDa appeared weak or disappeared, indicating that SP was degraded. It is likely that phosphofructokinase (82.4 kDa), phosphoglucoisomerase (60 kDa), creatine kinase (30–40 kDa), phosphotriose isomerase (28 kDa), and other proteins were degraded. A previous study reported similar trends in Chinese Cantonese sausages, where the degree of protein hydrolysis increased with processing time [34]. It has been demonstrated that both MP and SP are susceptible to temperature and proteases. A decrease in protease activity has been shown to inhibit protein degradation [33]. Therefore, in this study, it can be speculated that the increase in temperature and protease activity promotes protein hydrolysis. Protein degradation fragments have been shown to affect flavor and have important implications for interpreting flavor formation mechanisms [35]. Similarly, Li found that in the early stage of ham processing, some of the sarcoplasmic proteins degraded into smaller molecules and then underwent a series of biochemical reactions to produce the unique taste and flavor of the ham [9]. Therefore, the present study showed that the hydrolysis of MP and SP may be closely related to the formation of volatile flavor compounds.

An increase in protein degradation leads to differences in the content of free amino acids [36]; the levels of 24 free amino acids in bacon samples from different stages of LTS are presented in Table 3. Compared with control samples (0 d), the content of free amino acids increased significantly from 406.68 mg/100 g to 736.55 mg/100 g during LTS. With the exception of Cys and Gln, the content of most amino acids increased, which might have contributed to the formation of flavor compounds. It has been noted that amino acids such as Asp, Glu, Gly, Ala, Arg, and Leu are considered as precursors of meat aroma, which can react with soluble reducing sugars (e.g., glucose and fructose) to form flavors [37]. For example, Leu can react with the products of the Maillard reaction to form aldehydes, which significantly affects the volatile flavor of meat [38]. Meanwhile, the increase in free amino acid content in this study was accompanied by an increase in the content of flavor compounds, such as ketones and aldehydes, so it was hypothesized that the formation of characteristic flavor compounds of bacon during LTS is related to amino acid metabolism.

#### 3.4.2. Protein Oxidation

**Figure 3:**
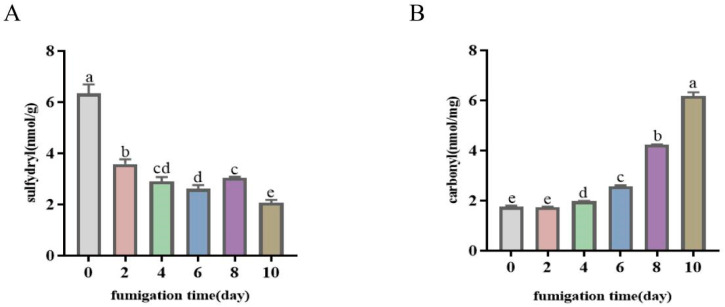
Protein oxidation in bacon during LTS. (**A**) The content of sulfhydryl groups in bacon during LTS. (**B**) The content of carbonyl groups in bacon during LTS. Results are expressed as mean ± standard derivation. ^a–e^ Means within rows and same breed with different superscripts differ significantly (*p* < 0.05).

Protein oxidation influences flavor development. The content of sulfhydryl groups, which form inter- and intramolecular disulfide links upon protein oxidation, decreases as the oxidation process progresses; this decrease serves as an indicator of the extent of protein oxidation in meat products [39]. Changes in the content of protein sulfhydryl groups in bacon during LTS are shown in Figure 3A. As expected, the total number of sulfhydryl groups significantly decreased from 6.34 nmol/g to 2.07 nmol/g (*p* < 0.05), indicating the occurrence of protein oxidation. The data clearly show that the duration of LTS had a substantial impact on protein oxidation in bacon. Moreover, these results align with those reported by Zhou et al. [40], who showed that protein oxidation led to a decrease in the sulfhydryl content in smoked bacon.

Additionally, the carbonyl content can be used to characterize the degree of protein oxidation. The decreasing trend in protein thiol groups was consistent with the formation of carbonyl compounds (Figure 3B), whose content increased from 1.76 nmol/mg to 6.18 nmol/mg (*p* < 0.05) by the end of LTS, indicating that protein oxidization continued to occur at this stage. This may be due to the continuous changes in processing temperature and catalysis by oxidases. In a study of Cantonese sausages, the same trend was observed regarding the carbonyl content during processing [41]. Soladoye et al. [42] reported a significant increase in the total carbonyl content of bacon during the smoking process and noted that the temperature during bacon processing was the main factor affecting the carbonyl content. It has also been suggested that proteolysis promotes protein oxidation in meat [43].

### 3.5. Qualitative and Quantitative Analyses of Changes in Bacon Proteins during LTS

**Figure 4:**
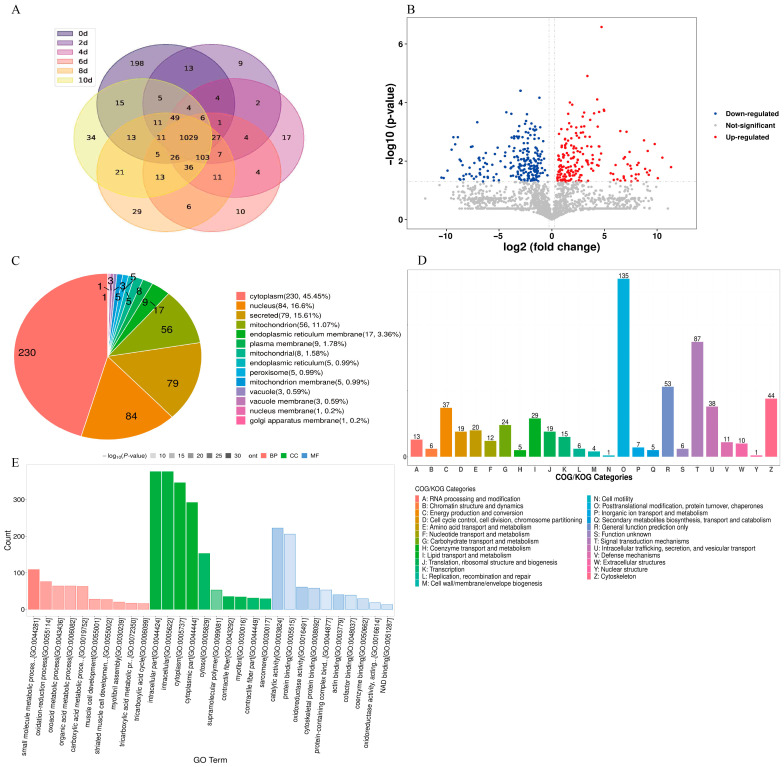
Protein expression and differentially abundant proteins in bacon during LTS. (**A**) Venn diagram. (**B**) Volcano plot of differentially abundant protein. (**C**) Pie chart of the subcellular localization of differentially abundant proteins. (**D**) Bar chart of KOG analysis; (**E**) GO analysis.

To further evaluate the protein changes induced by LTS, label-free proteomic analysis was performed. In total, 2017 proteins were quantified in the bacon samples, but the proteins differed across the different stages of LTS (Figure 4A), indicating that the types and contents of proteins in bacon changed markedly during LTS.

The key differentially abundant proteins (DAPs) during LTS were further screened using the following criteria: a *p*-value < 0.05 and an FC ≤ 0.83 or ≥1.2 with Student’s *t*-test or the chi-squared test. As shown in the volcano plot in Figure 4B, red dots represent significantly up-regulated DAPs, blue dots represent significantly down-regulated DAPs, and gray dots represent DAPs that changed insignificantly.

As shown in Figure 4A,B, the up-regulated DAPs in bacon during LTS comprised collagen alpha-1(I) chain preproprotein (A0A287A1S6), caveolae-associated protein 1 (A0A286ZZ97), myristoylated alanine-rich protein kinase C substrate (A0A287BRL8), protein S100-A11 (P31950), and cardiomyopathy-associated 5 (A0A286ZYS8), whereas the down-regulated DAPs comprised aconitate hydratase, mitochondrial (P16276), vitamin D-binding protein (A0A287AHK1), mannose-6-phosphate isomerase (A0A287BNZ5), CXXC motif-containing zinc-binding protein (F1S765), and LIM zinc-binding domain-containing protein (A0A287AYR8).

### 3.6. Bioinformatic Analysis of DAPs in Bacon during LTS

Bioinformatic analysis can help predict the subcellular location of proteins. As shown in Figure 4C, DAPs were distributed in the cytoplasm, nucleus, mitochondrion, endoplasmic reticulum, plasma membrane, vacuole, peroxisome, and Golgi apparatus and were most often found in the cytoplasm, nucleus, and mitochondrion as well as in secretions. During the LTS of bacon, the protein composition changes not only in the cytoplasm and cytoskeleton but also in the mitochondria, nucleus, and endoplasmic reticulum. The homologous classification of gene products via EuKaryotic Orthologous Groups analysis (Figure 4D) showed that some of these proteins were involved in several metabolic processes during duplication. They were mainly associated with metabolic functions, such as post-translational modifications, protein turnover, chaperones, signal transduction mechanisms, general function prediction only, cytoskeleton, intracellular trafficking, secretion, and vesicular transport.

To assess the impact of LTS on biological processes in bacon, the functional characteristics of DAPs at the beginning (0 d) and end (10 d) of LTS were analyzed using the Gene Ontology (GO) annotation method. The knowledge system and structural characteristics of GO annotations provide background knowledge, such as gene function classification tags or information from studies on the function of single-feature gene classes or combinations of multiple-feature function classes, which are associated with differentially expressed genes. GO annotations classified the proteins into three groups based on their functional activity: biological processes, molecular functions, and cellular components (Figure 4E). Among these, the proteins associated with cellular components were the most abundant.

### 3.7. Relationship between Protein Degradation or Oxidation and the Flavors of Bacon

**Figure 5:**
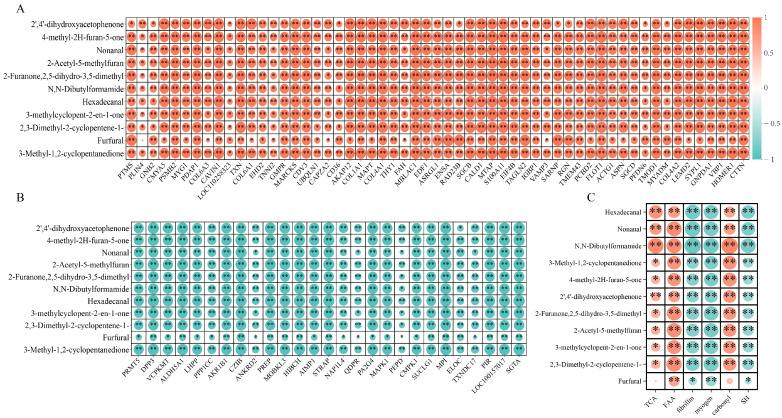
Correlation analysis between protein change and volatile flavor compounds. (**A**) Proteome positively associated with key volatile flavor compounds. (**B**) Proteome negatively associated with key volatile flavor compounds. (**C**) Correlation analysis of protein degradation and oxidation with key volatile flavor compounds. *: *p* < 0.05, **: *p* < 0.01.

To explore the relationship between DAPs and volatile compounds in bacon during LTS, DAPs were screened based on an FC of ≥3 or ≤0.1 and a *p*-value of <0.01. The correlation analysis (Figure 5A,B) revealed 57 positively correlated proteins and 27 negatively correlated proteins correlated with volatile compounds. Protein degradation was the main pathway for the formation of volatile compounds. Endopeptidases (cathepsin and calpain) degrade myofibrillar structures by breaking peptide bonds inside proteins, producing macromolecular polypeptides, whereas exopeptidases (aminopeptidases and tri- and dipeptidyl peptidases) further hydrolyze macromolecular polypeptides into smaller taste peptides and large amounts of free amino acids, which typically produce the flavor of the final product [44]. Additionally, 27 characteristic proteins were identified to be related to volatile compounds formed from protein degradation in the flavor compound formation pathway; the specific protein-related information is shown in Table 4. The main Kyoto Encyclopedia of Genes and Genomes (KEGG) pathways involved were metabolic pathways, carbon metabolism, propanoate metabolism, the TCA cycle, and amino acid catabolism. According to KEGG pathway enrichment analysis, amino acid metabolism pathways, including glutathione, cysteine, methionine, arginine, proline, alanine, aspartate, and glutamate metabolism pathways, were affected at every stage of LTS. The results of the KEGG analysis aligned with those of previous studies [6], showing that interactions with the TCA cycle can significantly affect purine and amino acid metabolisms, which ultimately impact flavor regulation.

Figure 5C shows the correlation analysis between the levels of characteristic volatile compounds and the degradation or oxidation of proteins, further demonstrating the relationship between protein and flavor compounds. The results indicated that volatile compounds were positively correlated with TCA and free amino acids and negatively correlated with the concentration of myofibrillar and sarcoplasmic proteins. This suggests that protein degradation plays a key role in flavor formation. It has been demonstrated that free amino acids are the primary compounds produced by protein hydrolysis and that the interactions between these compounds are the most critical factors leading to changes in volatile compounds [10]. This is consistent with the results of proteomics. This study found a positive correlation between volatile compounds and carbonyl content and a negative correlation between volatile compounds and total sulfhydryl content, aligning with the findings of previous analyses [45]. Carbonyl compounds, such as aldehydes and ketones, significantly influence the flavor of meat products, and these substances are mostly generated by protein oxidation [46].

## 4. Conclusions

In this study, 11 major volatile flavor compounds, including ketones (6), aldehydes (3), furans (1), and nitrides (1), were screened via HS-SPME-GC-MS in Hunan bacon during LTS. Results of proteomics showed that 27 proteins were identified to be associated with bacon flavor compounds, which mostly comprised endogenous enzymes (16) involved in protein degradation. Of these, seven were identified to be involved in amino acid metabolic pathways and metabolic processes. Changes in chemical parameters, including components of TCA-soluble peptides and free amino acid content, supported the proteomic analysis results. The correlation also suggests that protein oxidation may affect flavor to some extent. This study elucidates the relationship between degradation and the oxidation of protein and volatile flavor compounds in bacon during LTS. Lastly, although a proteomic methodology was used to investigate the formation of volatile flavor compounds in bacon during LTS in this study, further research is needed to confirm our proteomic analysis results.

## Figures and Tables

**Table 1:** Changes of physicochemical properties in the Hunan bacon during LTS.

Item	Hunan Bacon Low-Temperature Liquid Smoking Time (Days)					
	0	2	4	6	8	10
L*	48.3 ± 1.12 ^e^	59.5 ± 2.05 ^bc^	63.9 ± 1.36 ^a^	61.0 ± 2.14 ^ab^	54.7 ± 1.67 ^d^	57.0 ± 2.28 ^cd^
a*	4.91 ± 0.76 ^abc^	3.51 ± 1.38 ^cd^	3.10 ± 0.45 ^d^	5.73 ± 0.75 ^a^	3.55 ± 0.13 ^bcd^	5.08 ± 0.59 ^ab^
b*	5.28 ± 0.47 ^b^	5.33 ± 0.47 ^b^	7.39 ± 1.37 ^b^	10.84 ± 1.97 ^a^	6.77 ± 1.29 ^b^	10.3 ± 1.35 ^a^
Hardness	235 ± 75.4 ^c^	693 ± 135 ^b^	362 ± 135 ^bc^	675 ± 284 ^b^	1193 ± 283 ^a^	1219 ± 160 ^a^
Elasticity	0.86 ± 0.08	0.85 ± 0.02	0.81 ± 0.04	0.88 ± 0.08	0.95 ± 0.06	0.91 ± 0.08
Adhesiveness	211 ± 82.1 ^c^	682 ± 31.9 ^b^	351 ± 103 ^c^	364 ± 69.9 ^c^	958 ± 172 ^a^	959 ± 32.7 ^a^
Chewiness	157 ± 52.0 ^d^	584 ± 79.6 ^b^	344 ± 106 ^c^	241 ± 38.9 ^cd^	888 ± 162 ^a^	836 ± 41.2 ^a^
Resilience	0.19 ± 0.08 ^b^	0.20 ± 0.02 ^b^	0.16 ± 0.03 ^b^	0.21 ± 0.03 ^b^	0.31 ± 0.04 ^a^	0.32 ± 0.05 ^a^
Moisture	70.0 ± 1.56 ^a^	60.5 ± 1.73 ^b^	43.1 ± 0.39 ^d^	41.2 ± 1.67 ^de^	49.4 ± 1.59 ^c^	39.2 ± 1.75 ^e^

Results are expressed as mean ± standard derivation. ^a–e^ Means within rows and same breed with different superscripts differ significantly (*p* < 0.05, differences between fumigation time).

**Table 2:** Changes in the characteristic volatile flavor compounds of bacon during LTS.

Volatile Components (µg/kg)	VIP	CAS	Hunan Bacon Low-Temperature Liquid Smoking Time (Days)					
			0	2	4	6	8	10
2′,4′-dihydroxyacetophenone	1.02201	89-84-9	0	0	0.01 ± 0.01 ^c^	108 ± 10.6 ^a^	69.1 ± 26.3 ^b^	108 ± 14.2 ^a^
4-methyl-2H-furan-5-one	1.03731	22122-36-7	0	0	0.01 ± 0.02 ^b^	193 ± 27.6 ^a^	181 ± 11.9 ^a^	200 ± 29.4 ^a^
Nonanal	1.07986	124-19-6	6.58 ± 4.93 ^a^	34.2 ± 8.54 ^ab^	58.9 ± 5.55 ^b^	137 ± 41.5 ^a^	111 ± 12.5 ^a^	133 ± 24.9 ^a^
2-Acetyl-5-methylfuran	1.12646	1193-79-9	0.11 ± 0.05 ^b^	5.02 ± 0.79 ^b^	35.9 ± 5.15 ^b^	254 ± 16.1 ^a^	242 ± 34.1 ^a^	244 ± 35.8 ^a^
2-Furanone,2,5-dihydro-3,5-dimethyl	1.26189		0.02 ± 0.03 ^b^	18.9 ± 2.70 ^b^	48.1 ± 3.75 ^b^	353 ± 48.6 ^a^	342 ± 24.6 ^a^	338 ± 64.2 ^a^
*N*,*N*-Dibutylformamide	1.39613	761-65-9	0	22.6 ± 3.91 ^cd^	18.3 ± 1.86 ^d^	33.1 ± 5.48 ^bc^	37.1 ± 6.01 ^b^	59.5 ± 10.9 ^a^
Hexadecanal	1.66915	629-80-1	1.63 ± 0.65 ^c^	30.6 ± 3.16 ^c^	98.2 ± 21.19 ^b^	241 ± 34.8 ^a^	107 ± 6.75 ^b^	245 ± 32.3 ^a^
3-methylcyclopent-2-en-1-one	1.6924	2758-18-1	0	0.30 ± 0.23 ^b^	72.0 ± 21.7 ^b^	537 ± 91.7 ^a^	513 ± 34.5 ^a^	511 ± 100 ^a^
2,3-Dimethyl-2-cyclopentene-1-one	1.7476	1121-05-7	0	51.5 ± 2.27 ^b^	109 ± 7.54 ^b^	561 ± 43.3 ^a^	570 ± 76.9 ^a^	546 ± 105 ^a^
Furfural	2.372	98-01-1	0.62 ± 0.41 ^c^	1.18 ± 0.70 ^c^	0.46 ± 0.37 ^c^	735 ± 189 ^a^	672 ± 33.5 ^a^	337 ± 115 ^b^
3-Methyl-1,2-cyclopentanedione	2.88217	765-70-8	38.6 ± 1.26 ^b^	62.3 ± 3.70 ^b^	63.6 ± 4.27 ^b^	1553 ± 95.9 ^a^	1569 ± 289 ^a^	1592 ± 217 ^a^

Results are expressed as mean ± standard derivation. ^a–d^ Means within rows and same breed with different superscripts differ significantly (*p* < 0.05, differences between fumigation time).

**Table 3:** Changes in the level of free amino acids in bacon during LTS.

FAA (mg/100 g)	Hunan Bacon Low-Temperature Liquid Smoking Time (Days)					
	0	2	4	6	8	10
Asp	0.84 ± 0.54 ^cd^	0.76 ± 0.05 ^d^	1.35 ± 0.36 ^c^	2.54 ± 0.07 ^b^	2.61 ± 0.26 ^b^	3.21 ± 0.12 ^a^
Glu	9.60 ± 0.78 ^e^	22.9 ± 2.11 ^d^	28.7 ± 2.11 ^c^	40.8 ± 0.99 ^a^	35.9 ± 3.38 ^b^	41.3 ± 0.72 ^a^
Asn	2.76 ± 0.27 ^d^	5.15 ± 0.39 ^c^	7.79 ± 0.57 ^a^	6.20 ± 0.49 ^b^	7.54 ± 0.69 ^a^	8.22 ± 0.16 ^a^
Ser	5.86 ± 0.46 ^e^	12.0 ± 0.98 ^d^	16.9 ± 1.24 ^c^	19.2 ± 0.77 ^b^	19.6 ± 0.97 ^b^	21.9 ± 0.40 ^a^
Gln	28.5 ± 1.90 ^a^	29.4 ± 2.81 ^a^	19.8 ± 1.46 ^b^	14.0 ± 0.34 ^c^	14.9 ± 1.51 ^c^	7.92 ± 0.44 ^d^
His	2.12 ± 0.27 ^e^	4.23 ± 0.48 ^d^	5.67 ± 0.44 ^cd^	6.33 ± 0.28 ^bc^	7.45 ± 1.12 ^ab^	8.13 ± 1.54 ^a^
Gly	8.98 ± 0.45 ^e^	14.7 ± 1.29 ^d^	17.9 ± 1.37 ^c^	21.1 ± 0.81 ^b^	23.3 ± 0.93 ^ab^	25.0 ± 1.22 ^a^
Thr	5.48 ± 0.73 ^e^	9.99 ± 1.12 ^d^	13.4 ± 0.93 ^c^	16.2 ± 0.79 ^b^	16.6 ± 1.18 ^b^	18.9 ± 1.29 ^a^
Cit	1.77 ± 0.81 ^c^	2.27 ± 0.70 ^c^	3.55 ± 0.22 ^c^	8.61 ± 0.93 ^b^	7.94 ± 3.75 ^b^	13.6 ± 1.88 ^a^
Arg	5.48 ± 0.59 ^d^	11.1 ± 1.55 ^c^	16.9 ± 1.29 ^b^	18.9 ± 0.80 ^b^	19.5 ± 2.50 ^b^	23.4 ± 1.88 ^a^
Ala	168 ± 13.0 ^d^	201 ± 18.7 ^c^	214 ± 17.7 ^bc^	208 ± 5.64 ^bc^	256 ± 11.1 ^a^	227 ± 4.20 ^b^
Tyr	3.73 ± 0.37 ^c^	7.79 ± 0.89 ^b^	10.31 ± 0.82 ^a^	10.9 ± 0.45 ^a^	11.0 ± 0.73 ^a^	10.9 C ± 0.15 ^a^
Cys	1.90 ± 1.44	1.72 ± 2.32	0.49 ± 0.05	0.51 ± 0.02	3.69 ± 5.50	0.49 ± 0.02
Val	5.42 ± 0.33 ^e^	11.0 ± 0.60 ^d^	14.9 ± 1.14 ^c^	17.6 ± 0.49 ^b^	18.1 ± 1.21 ^b^	21.1 ± 0.34 ^a^
Met	4.86 ± 0.30 ^c^	7.84 ± 0.62 ^b^	9.73 ± 0.75 ^a^	9.62 ± 0.28 ^a^	10.5 ± 0.48 ^a^	10.4 ± 0.26 ^a^
Nva	69.8 ± 4.73 ^d^	77.0 ± 5.64 ^cd^	83.2 ± 5.65 ^bc^	77.3 ± 2.64 ^cd^	111 ± 6.72 ^a^	89.5 ± 0.61 ^b^
Trp	1.34 ± 0.14 ^b^	2.19 ± 0.28 ^a^	2.65 ± 0.24 ^a^	2.79 ± 0.14 ^a^	2.27 ± 0.97 ^a^	2.84 ± 0.05 ^a^
Phe	8.07 ± 0.60 ^d^	13.6 ± 1.10 ^c^	19.1 ± 1.45 ^b^	19.4 ± 0.61 ^b^	20.8 ± 1.27 ^ab^	22.3 ± 0.44 ^a^
Ile	4.80 ± 0.34 ^e^	9.95 ± 0.82 ^d^	13.9 ± 1.02 ^c^	15.3 ± 0.40 ^bc^	15.7 ± 0.96 ^b^	17.3 ± 0.28 ^a^
Leu	7.44 ± 0.58 ^e^	15.2 ± 1.40 ^d^	25.5 ± 1.88 ^c^	27.9 ± 0.66 ^bc^	29.4 ± 1.56 ^b^	32.1 ± 0.58 ^a^
Lys	6.79 ± 0.56 ^e^	18.2 ± 1.76 ^d^	26.2 ± 2.00 ^c^	31.7 ± 0.76 ^b^	32.7 ± 1.86 ^b^	38.5 ± 0.75 ^a^
Hyp	40.7 ± 3.00 ^d^	48.9 ± 3.93 ^c^	54.1 ± 4.33 ^bc^	53.4 ± 1.78 ^bc^	56.5 ± 1.39 ^b^	58.2 ± 1.93 ^ab^
Sar	9.69 ± 0.65 ^d^	12.1 ± 1.21 ^c^	15.0 ± 1.00 ^b^	15.4 ± 0.59 ^b^	17.4 ± 0.76 ^a^	17.3 ± 0.39 ^a^
Pro	2.48 ± 0.78 ^e^	6.91 ± 2.03 ^d^	9.79 ± 1.16 ^cd^	13.6 ± 0.88 ^ab^	12.1 ± 1.26 ^bc^	16.8 ± 2.05 ^a^
FAA	407 ± 27.9 ^d^	546 ± 47.1 ^c^	630 ± 45.8 ^b^	657 ± 17.6 ^b^	752 ± 5.67 ^a^	737 ± 19.0 ^a^

Results are expressed as mean ± standard derivation. ^a–e^ Means within rows and same breed with different superscripts differ significantly (*p* < 0.05, differences between fumigation time).

**Table 4:** Information of flavor-related protein in bacon during LTS.

Gene Name	Accession	Protein
PRMT5	A0A5G2QRI8	Protein arginine N-methyltransferase 5
DPP3	F1RU52	Dipeptidyl peptidase 3
VCPKMT	A0A287B739	Valosin-containing protein lysine methyltransferase
ALDH5A1	F1RUE3	Succinate-semialdehyde dehydrogenase
LHPP	A0A480STU2	Phospholysine phosphohistidine inorganic pyrophosphate phosphatase
PPP1CC	Q2EHH7	Serine/threonine-protein phosphatase
AKR1B1	P80276	Aldo-keto reductase family 1 member B1
CZIB	F1S765	CXXC motif-containing zinc-binding protein
ANKRD2	A0A5G2QKX2	Ankyrin repeat domain 2
PREP	P23687	Prolyl endopeptidase
MOBKL3	F2Z5T8	MOB-like protein phocein
HIBCH	A0A5G2QLF4	3-hydroxyisobutyryl-CoA hydrolase
AIMP1	A0A287AKA2	Aminoacyl tRNA synthetase complex interacting multifunctional protein 1
STRAP	A0A4X1VAU7	Serine-threonine kinase receptor-associated protein
NAP1L4	A0A481CQ30	Nucleosome assembly protein 1-like 4
QDPR	A0A5G2QZN1	Quinoid dihydropteridine reductase
PA2G4	A0A8D0X235	Peptidase M24 domain-containing protein
MAPK1	A0A8W4FG96	Mitogen-activated protein kinase
PEPD	A0A5G2QM19	Peptidase D
CMPK1	Q29561	UMP-CMP kinase
SUCLG1	O19069	Succinate—CoA ligase [ADP/GDP-forming] subunit alpha, mitochondrial
MPI	A0A287BNZ5	Mannose-6-phosphate isomerase
ELOC	A0A8D0P6A0	Elongin-C
TXNDC17	A0A286ZZM7	Thioredoxin domain-containing protein 17
PIR	K7GKW6	Pirin
LOC100157017	A0A287A5X0	Glyoxylate reductase/hydroxypyruvate reductase
SGTA	A0A287A1V6	Small glutamine-rich tetratricopeptide repeat co-chaperone alpha

## Data Availability

The original contributions presented in the study are included in the article, further inquiries can be directed to the corresponding authors.

## Supplementary Materials

The following supporting information can be downloaded at: https://www.mdpi.com/article/10.3390/foods13091360/s1, Table S1: Changes in volatile flavor compounds of bacon during LTS.
