# Supplementary material for: Quantitative Proteomics Reveals the Relationship between Protein Changes and Volatile Flavor Formation in Hunan Bacon during Low-Temperature Smoking
Source: Foods. 2024 Apr 28;13(9):1360. doi: 10.3390/foods13091360 (PMC11083045; doi:10.3390/foods13091360)
Supplement: Supplementary file 1 [file foods-13-01360-s001.zip › foods-2972067-supplementary.pdf]

foods MDPI

Table S1. Changes in volatile flavor compounds of bacon during LTS.

	Volatile compounds (μg/kg)	CAS	0	2	4	6	8	10
Aldehydes	Furfural	98-01-1	0.62±0.41^{c}	1.18±0.70^{c}	0.46±0.37^{c}	735±189^{a}	672±33.5^{a}	337±115^{b}
	Nonanal	124-19-6	6.58±4.93^{a}	34.2±8.54^{ab}	58.9±5.55^{b}	137±41.5^{a}	111±12.5^{a}	133±24.9^{a}
	Decanal	112-31-2	2.40±0.99^{c}	3.20±0.76^{c}	3.91±0.92^{c}	9.76±3.54^{ab}	13.2±5.55^{a}	7.17±1.46^{bc}
	Tetradecanal	124-25-4	0.69±0.43^{b}	2.02±0.68^{b}	2.83±0.24^{b}	6.71±0.79^{a}	8.17±1.38^{a}	8.24±2.44^{a}
	Pentadecanal	2765-11-9	2.03±1.88^{c}	5.93±4.35^{abc}	3.98±0.86^{bc}	10.3±1.53^{a}	8.33±2.32^{ab}	9.73±2.03^{a}
	Hexadecanal	629-80-1	1.63±0.65^{c}	30.6±3.16^{c}	98.2±21.2^{b}	241±34.8^{a}	107±6.75^{b}	245±32.3^{a}
	Octadecanal	638-66-4	0.48±0.11^{e}	2.75±0.50^{d}	5.61±1.13^{c}	14.1±2.05^{a}	8.33±0.70^{b}	14.7±5.56^{a}
Ketones	Dihydropyran-3-one	23462-75-1	0.30±0.12^{b}	2.98±1.30^{b}	5.08±1.25^{b}	42.1±8.66^{a}	38.9±1.39^{a}	38.7±5.91^{a}
	3-methylcyclopent-2-en-1-one	2758-18-1	0	0.30±0.23^{b}	72.0±21.7^{b}	537±91.7^{a}	513±34.5^{a}	511±100^{a}
	4-methyl-2H-furan-5-one	22122-36-7	0	0	0.01±0.02^{b}	193±27.6^{a}	181±11.9^{a}	200±29.4^{a}
	3-methylcyclohex-3-en-1-one	31883-98-4	0	7.58±0.14^{b}	17.6±0.41^{b}	116±17.5^{a}	99.5±7.98^{a}	94.9±21.1^{a}
	2-Furanone, 2,5-dihydro-3,5-dimethyl		0.02±0.03^{b}	18.9±2.70^{b}	48.1±3.75^{b}	353±48.6^{a}	342±24.6^{a}	338±64.2^{a}
	3-Methyl-1,2-cyclopentanedione	765-70-8	38.6±1.26^{b}	62.3±3.70^{b}	63.6±4.27^{b}	1553±95.9^{a}	1569±289^{a}	1592±217^{a}
	2,3-Dimethyl-2-cyclopentene-1-one	1121-05-7	0	51.5±2.27^{b}	109±7.54^{b}	561±43.3^{a}	570±76.9^{a}	546±105^{a}
	3-methylcyclopentane-1,2,4-trione	4505-54-8	0	10.6±0.43^{b}	21.0±0.81^{b}	123±21.9^{a}	113±26.8^{a}	99.0±22.0^{a}
	4,5-dimethylhex-4-en-3-one	17325-90-5	0	7.48±1.68^{b}	19.9±2.08^{b}	136±31.4^{a}	132±28.2^{a}	128±18.58^{a}
	phenylacetone	103-79-7	0.01±0.02^{d}	0.20±0.10^{d}	2.94±0.60^{c}	16.1±1.15^{a}	12.9±2.16^{b}	12.6±1.87^{b}
	2-ethyl-3-methyl-2-cyclopenten-1-one	5682-72-4	0	5.72±0.46^{b}	12.3±0.96^{b}	79.7±11.1^{a}	71.7±11.9^{a}	66.6±9.67^{a}
	2',4'-dihydroxyacetophenone	89-84-9	0	0	0.01±0.01^{c}	108±10.59^{a}	69.1±26.3^{b}	108±14.2^{a}
	3,4-dimethyl-2H-furan-5-one	1575-46-8	0.29±0.16^{c}	0.42±0.26^{c}	13.2±0.47^{c}	145±16.4^{a}	121±17.8^{b}	136±15.7^{ab}
	dihydro-3-methyl-2(3H)-Furanone	1679-47-6	0.26±0.13^{b}	0.33±0.31^{b}	6.03±1.83^{b}	26.3±4.95^{a}	28.8±4.74^{a}	22.6±3.53^{a}
Esters	Methyl n-caprate	110-42-9	0	1.04±0.14^{c}	1.72±0.25^{c}	7.61±0.66^{b}	10.6±1.27^{a}	8.75±1.88^{ab}
	Methyl hexadecanoate	112-39-0	0.47±0.34^{b}	0.73±0.31^{b}	1.36±0.36^{b}	6.88±4.43^{a}	3.75±1.31^{ab}	7.48±1.38^{a}
	dibutyl benzene-1,4-dicarboxylate	1962-75-0	33.6±27.9^{ab}	11.8±13.5^{b}	53.3±83.5^{ab}	239±205^{a}	68.8±43.3^{ab}	157±78.2^{ab}
Acids	2-methylbutyric acid	116-53-0	0	1.44±0.63^{b}	4.89±1.13^{b}	52.7±11.3^{a}	43.8±3.33^{a}	44.7±10.5^{a}
	valeric acid	109-52-4	0.01±0.01^{b}	0.46±0.15^{b}	0.86±1.08^{b}	67.3±15.5^{a}	47.1±9.65^{b}	56.5±10.14^{ab}
	Octanoic acid	124-07-2	0	0.02±0.02^{c}	2.20±1.80^{c}	50.2±5.01^{ab}	41.0±11.9^{b}	53.1±5.01^{a}
	Decanoic acid	334-48-5	0	0	0.01±0.02^{b}	14.0±3.99^{a}	15.2±5.32^{a}	13.1±2.11^{a}
	Palmitic acid	57-10-3	2.70±0.06^{b}	9.40±6.30^{b}	9.49±6.57^{b}	44.0±32.6^{a}	22.5±1.45^{ab}	17.1±5.68^{ab}
Furans	2-Acetyl-5-methylfuran	1193-79-9	0.11±0.05^{b}	5.02±0.79^{b}	35.9±5.15^{b}	254±16.1^{a}	242±34.1^{a}	244±35.8^{a}
Alkanes	Limonene	138-86-3	0.84±0.05^{c}	3.48±0.86^{b}	1.17±0.21^{c}	7.53±0.56^{a}	8.15±1.14^{a}	3.84±1.34^{b}
	5-methyldecane	13151-35-4	1.55±0.28^{b}	1.80±0.22^{b}	4.64±0.12^{b}	23.1±3.00^{a}	26.8±6.95^{a}	21.9±4.68^{a}
	Dodecane	112-40-3	4.73±1.68^{b}	5.38±1.35^{b}	5.20±0.82^{b}	12.8±2.72^{a}	12.1±2.81^{a}	5.68±1.64^{b}
	2,6,11-Trimethyldodecane	31295-56-4	4.41±2.10^{bc}	3.79±0.66^{c}	3.19±0.42^{c}	8.41±3.46^{ab}	10.2±2.62^{a}	4.64±0.78^{bc}
	Tetradecane	629-59-4	2.97±0.50^{c}	4.22±1.12^{c}	4.38±0.74^{c}	24.3±5.59^{b}	33.6±6.42^{a}	24.8±2.56^{b}

	Pentadecane	629-62-9	1.10±0.26[c]	2.84±0.13[c]	3.55±0.49[c]	29.8±4.31[b]	31.0±9.45[b]	40.2±3.45[a]
	Hexadecane	544-76-3	3.18±1.48[c]	2.14±0.27[c]	3.99±2.33[c]	14.5±3.70[b]	16.3±3.33[b]	22.4±2.50[a]
	Heptadecane	629-78-7	1.24±0.12[b]	1.64±0.13[b]	1.73±0.14[b]	6.32±1.57[a]	5.25±0.12[a]	5.66±0.89[a]
	Eicosane	112-95-8	0.56±0.52[c]	2.32±0.34[c]	5.25±1.2[b]	13.2±1.38[a]	7.8±2.6[b]	13.5±1.32[a]
	1,2,3-Trimethoxybenzene	634-36-6	0	0.99±0.10[c]	2.72±0.44[c]	31.8±6.75[a]	22.8±3.11[b]	26.3±4.02[ab]
	3-methylbut-2-enylbenzene	4489-84-3	0.01±0.01[b]	0.62±0.82[b]	2.82±0.36[b]	22.2±3.83[a]	18.1±2.41[a]	19.1±2.71[a]
Nitrides	N,N-Dibutylformamide	761-65-9	0	22.6±3.91[cd]	18.3±1.86[d]	33.1±5.48[bc]	37.1±6.01[b]	59.5±10.9[a]

Results were expressed as mean ± standard derivation. [a-e] Means within rows and same breed with different superscripts differ significantly ($P < 0.05$, differences between fumigation time).
